# Predictors of subsequent relapse-independent progression after high-efficacy therapy in relapsing multiple sclerosis

**DOI:** 10.3389/fimmu.2026.1935407

**Published:** 2026-08-19

**Authors:** Matteo Foschi, Andrea Surcinelli, Ivan Panzera, Ludovica Maria Miserocchi, Giorgia Balducci, Luca Mancinelli, Cristiana Ganino, Francesca Graziano, Ester Lagonigro, Maria Di Ioia, Valeria Di Tommaso, Deborah Farina, Giovanna De Luca, Massimo Caulo, Maria Grazia Piscaglia, Stefano Luca Sensi, Valentina Tomassini

**Affiliations:** 1 Multiple Sclerosis Center - Neurology Unit, Department of Neurosciences, S. Maria delle Croci Hospital, AUSL Romagna, Ravenna, Italy; 2 Multiple Sclerosis Center - Neurology Unit, Department of Neurosciences, Maurizio Bufalini Hospital, AUSL Romagna, Cesena, Italy; 3 Multiple Sclerosis Center - Neurology Unit, Department of Neurosciences, Infermi Hospital, AUSL Romagna, Rimini, Italy; 4 Institute of Advanced Biomedical Technologies (ITAB), Department of Neurosciences, Imaging and Clinical Sciences, University G. d’Annunzio of Chieti-Pescara, Chieti, Italy; 5 Center for Disability, Rehabilitation and Sports Medicine (CARES), Department of Neuroscience and Clinical Sciences, University of Chieti, Chieti, Italy; 6 Department of Neurosciences, Imaging and Clinical Sciences, University G. d’Annunzio of Chieti-Pescara, Chieti, Italy; 7 Multiple Sclerosis (MS) Centre, Neurology Institute, SS. Annunziata University Hospital, Chieti, Italy

**Keywords:** age at onset, disease-modifying therapies, high-efficacy therapy, multiple sclerosis, progression independent of relapse activity, relapse-associated worsening, predictors

## Abstract

**Background and objectives:**

Progression independent of relapse activity (PIRA) is increasingly recognized as a major driver of disability accumulation in multiple sclerosis (MS). However, the factors associated with PIRA occurring after initiation of high-efficacy therapies (HETs) remain poorly investigated. We aimed to identify clinical predictors of subsequent PIRA following first HET initiation.

**Methods:**

We retrospectively analyzed data from two prospectively maintained Italian MS registries. Adults with relapsing MS who initiated a HET and had at least one year of post-treatment follow-up were included. Cox proportional hazards models were used to identify predictors of PIRA after first HET initiation. A primary multivariable model included age at disease onset, sex, baseline Expanded Disability Status Scale (EDSS) score, time from symptom onset to HET initiation, and annualized relapse rate (ARR) before HET exposure. A secondary model additionally adjusted for baseline brain MRI characteristics. Exploratory analyses included risk stratification according to age and treatment timing, strategy-specific analyses, comparison of predictors of PIRA and relapse-associated worsening (RAW), risk of PIRA across first HET classes, and sensitivity analyses.

**Results:**

Among 466 patients with relapsing MS (71.5% female, median age at onset 32.2 years), 70 (15.0%) developed PIRA after first HET initiation. In the primary multivariable model, older age at onset (HR 1.07, 95% CI 1.05–1.10; p<0.001) and longer time from symptom onset to HET initiation (HR 1.10, 95% CI 1.05–1.16; p<0.001) independently predicted PIRA. These associations remained consistent in the complete-case MRI-adjusted model including baseline brain MRI characteristics (n=269). Patients with younger age at onset and HET initiation within two years of symptom onset had the lowest risk of PIRA. In exploratory analyses, older age at onset and delayed HET initiation predicted PIRA but not RAW, whereas higher pre-treatment relapse activity predicted RAW but not PIRA. Findings were consistent across strategy-specific, on-treatment, landmark, EDSS re-baselining, and calendar-year-adjusted analyses.

**Conclusions:**

Older age at disease onset and delayed HET initiation were the most consistent predictors of PIRA. Our findings support the importance of early HET and suggest that clinically defined PIRA and relapse-associated worsening have partially distinct predictor profiles, while not excluding a contribution of subclinical MRI inflammatory activity to PIRA events.

## Introduction

Multiple sclerosis (MS) has traditionally been viewed as a disease in which disability accumulation is primarily driven by clinical relapses and incomplete recovery (1). However, clinical trials and real-world studies have shown that a substantial proportion of disability worsening occurs independently of relapses (2, 3). This clinically defined construct, termed progression independent of relapse activity (PIRA), does not require the absence of concurrent or recent magnetic resonance imaging (MRI) inflammatory activity and should not be equated with purely neurodegenerative progression (1–3). Nevertheless, PIRA is a major contributor to long-term disability accrual, including during the relapsing phase of MS (4–7).

Large observational cohorts and clinical trial populations have shown that PIRA accounts for most confirmed disability accumulation events and may emerge early after disease onset (4–7). These findings suggest that progression-related mechanisms are active from the earliest disease stages and may not be fully captured by conventional measures of inflammatory activity (8), making prevention of disability accumulation a therapeutic goal beyond relapse suppression alone (9).

High-efficacy therapy (HET) has substantially improved control of focal inflammatory activity, reducing relapse rates and MRI lesion formation (10). Nevertheless, disability worsening continues to occur in some treated patients, frequently as PIRA (2). Identifying predictors of PIRA after HET initiation may therefore improve risk stratification, treatment individualization, and therapeutic timing, while clarifying the relative contribution of inflammatory and non-inflammatory mechanisms.

Although predictors of disability worsening and PIRA have been investigated in heterogeneous MS populations (5–7, 11), data specifically addressing PIRA after HET initiation remain limited. It is unclear whether readily available clinical characteristics, including age at onset and timing of HET initiation, are more informative than baseline MRI measures, and whether PIRA and relapse-associated worsening (RAW) have distinct predictors. We therefore aimed to identify clinical predictors of subsequent PIRA after first HET initiation in a multicenter real-world cohort of patients with relapsing MS.

## Methods

### Data source

Data were obtained from two MS registries maintained at tertiary referral centers in Italy: Ravenna (S. Maria delle Croci Hospital, AUSL Romagna) and Chieti (S.S. Annunziata Hospital). Established in 2021 and 2016, respectively, the registries systematically collect demographic, clinical, radiological, disability, and treatment-related data from all patients with MS followed at the participating centers. Patients already under clinical care before registry implementation were retrospectively included through structured review of medical records, whereas subsequent patients were enrolled prospectively. Clinical assessments, Expanded Disability Status Scale (EDSS) evaluations, treatment information, and MRI findings were recorded during routine neurological visits and entered into harmonized electronic databases. Data for the present analysis were extracted on March 18, 2026.

### Ethics approval and consent to participate

The Ravenna MS registry received ethical approval from the local ethical committee [Comitato Etico della Romagna (CEROM)] on date 05/11/2021 (approval code: 3139). The Chieti MS registry was approved by the local ethical committee (Comitato Etico delle Province di Chieti e Pescara) on date 11/11/2016 (approval code: 20). All participants gave written informed consent to be enrolled in both registries according to the Declaration of Helsinki, 1964.

### Study design and population

This retrospective cohort study included adults with MS diagnosed according to the version of the McDonald criteria applicable at the time of diagnosis (2001, 2005, 2010, or 2017 revisions (12–15)). Eligible patients were required to have: (i) symptom onset between 2001 and 2024; (ii) age ≥18 years at symptom onset; (iii) an EDSS assessment within 12 months of symptom onset; (iv) at least two neurological evaluations performed ≥6 months apart; (v) a minimum follow-up duration of 12 months; and (vi) a relapsing MS phenotype at the time of first HET initiation. The 2001 lower boundary for symptom onset was chosen to align the cohort with the first McDonald criteria (12). Patients were included only if they subsequently initiated a HET while retaining a relapsing phenotype and had at least one year of post-HET follow-up, whereas the upper boundary of 2024 ensured adequate follow-up for outcome ascertainment.

The baseline reference EDSS score was defined as the first EDSS assessment performed at least one month after a relapse and subsequently confirmed after six months. Baseline MRI characteristics were derived from brain MRI examinations performed within ±12 months of the baseline clinical visit. When multiple brain MRI examinations were available, the scan closest to the baseline clinical visit was selected.

### Study outcomes

Confirmed disability accumulation (CDA) was defined as a sustained increase in EDSS score confirmed after at least six months, corresponding to an increase of ≥1.5 points for baseline EDSS = 0, ≥1.0 point for baseline EDSS 1.0–5.5, and ≥0.5 points for baseline EDSS >5.5. The date of CDA was defined as the first assessment documenting disability worsening (8).

CDA events were classified as either PIRA or RAW according to their temporal relationship with clinical relapses. PIRA was defined as CDA occurring in the absence of clinical relapses from 30 days before to 90 days after disability worsening (8). RAW was defined as CDA occurring within a relapse-associated period extending from 30 days before to 90 days after relapse onset (8). Thus, PIRA and RAW were mutually exclusive at the level of each disability worsening event. However, because patients could experience more than one disability worsening event during follow-up, PIRA and RAW were not considered mutually exclusive at the patient level. Because longitudinal brain and spinal MRI examinations temporally aligned with each disability-worsening event were not systematically available, MRI activity was not incorporated into event classification. Accordingly, the outcome analyzed in this study represents clinically defined PIRA rather than progression independent of both relapse and MRI activity.

### Statistical analysis

Continuous variables are presented as median and interquartile range (IQR), and categorical variables as counts and percentages. Baseline characteristics were summarized for the overall cohort and according to the occurrence of PIRA after initiation of the HET. Group comparisons were performed using the Wilcoxon rank-sum test for continuous variables and Pearson’s chi-square or Fisher’s exact tests for categorical variables, as appropriate.

In the primary analysis, patients entered the risk set at first HET initiation and were followed according to an intention-to-treat-like approach based on HET exposure. Accordingly, no pre-HET observation time or disability event contributed to the primary time-to-event analysis of PIRA after HET exposure. Time-to-event was calculated from HET initiation to the first confirmed PIRA event or censoring at the last available clinical follow-up, irrespective of subsequent treatment discontinuation or switching.

Predictors of PIRA were evaluated using Cox proportional hazards regression models. Univariable analyses were performed for demographic, clinical, treatment-related, and brain MRI variables. The primary multivariable model included age at disease onset, sex, baseline EDSS score, time from symptom onset to HET initiation, and annualized relapse rate (ARR) before HET exposure. Age at onset and time from symptom onset to HET initiation were included as separate prespecified variables to distinguish the association of age-related disease characteristics from that of treatment timing; age at HET initiation was therefore not entered as an additional covariate. A secondary model additionally incorporated baseline MRI characteristics, including gadolinium-enhancing lesions and high T2 lesion burden (≥10 lesions). MRI-adjusted analyses were performed as complete-case analyses and were restricted to patients with complete data for all clinical covariates and both prespecified brain MRI variables. Multicollinearity was evaluated using variance inflation factors (VIFs), the proportional hazards assumption using Schoenfeld residuals, and model discrimination using Harrell’s concordance index (C-index). Hazard ratios (HRs) with corresponding 95% confidence intervals (CIs) are reported.

Exploratory analyses included strategy-specific models (first-line HET vs switcher/escalation strategy), risk stratification according to age at onset and timing of HET initiation, restricted cubic spline modeling of continuous predictors, an exploratory comparison of PIRA across HET classes, and an event-context analysis comparing predictors of PIRA, RAW, and any CDA event.

Sensitivity analyses were performed to assess the robustness of the primary findings. First, an on-treatment analysis censored follow-up at discontinuation of the index HET or initiation of a subsequent disease-modifying therapy, whichever occurred first, to evaluate the influence of treatment persistence on the observed associations. Second, a landmark analysis introducing a 90-day lag period after HET initiation was performed to minimize potential reverse causation and the influence of early events that might reflect pre-existing disease activity rather than treatment response. Third, EDSS was re-baselined at first HET initiation using the most recent relapse-free EDSS assessment obtained within the preceding 180 days. Post-HET confirmed disability accumulation and PIRA events were then re-derived using this treatment-specific EDSS reference, and the primary clinical and MRI-adjusted models were repeated. Fourth, an additional sensitivity analysis included calendar year of first HET initiation as a continuous covariate in both the primary clinical and MRI-adjusted Cox models, to account for potential secular changes in HET availability, treatment selection, and clinical practice over the study period.

Given the observational design, no *a priori* sample size calculation was performed. All analyses were conducted using R version 4.2.2 (R Foundation for Statistical Computing, Vienna, Austria), with statistical significance defined as a two-sided p-value <0.05.

## Results

### Study cohort and baseline characteristics

**Figure 1:**
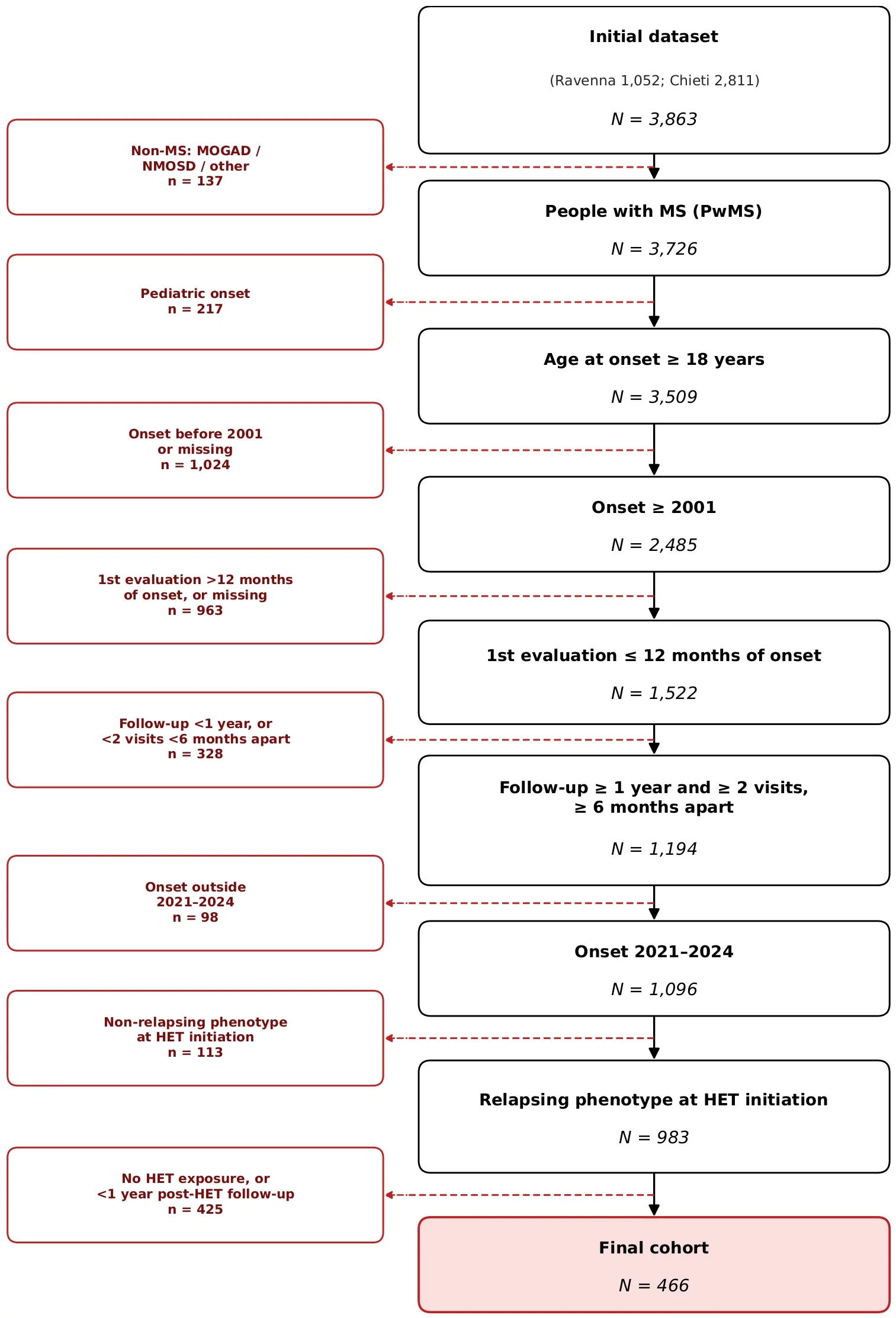
Flow chart of patient selection and derivation of the study cohorts. Patients were identified from the Ravenna (n=1,052) and Chieti (n=2,811) multiple sclerosis registries. Exclusion criteria were applied sequentially and included alternative diagnoses (NMOSD, MOGAD, or other non-MS conditions), pediatric-onset disease (<18 years), symptom onset before 2001, first neurological evaluation occurring more than 12 months after symptom onset, follow-up shorter than 1 year, and diagnosis after December 31, 2024. The resulting cohort comprised 1,137 patients. Patients were then classified according to disease phenotype at the time of high-efficacy therapy (HET) initiation. Individuals with a non-relapsing phenotype at HET initiation were excluded. Among patients with a relapsing phenotype at HET initiation, those who initiated HET and had at least one year of post-HET follow-up constituted the final analytical cohort (n=466). HET, high-efficacy therapy; MOGAD, myelin oligodendrocyte glycoprotein antibody-associated disease; MS, multiple sclerosis; NMOSD, neuromyelitis optica spectrum disorder; pwMS, people with multiple sclerosis.

The patient selection process is summarized in **Figure 1**. The final parent cohort consisted of 1,137 patients. Among these, 466 patients who initiated a HET and had at least one year of post-treatment follow-up were included in the present analysis.

Baseline demographic, clinical, and neuroimaging characteristics are reported in **Table 1**. The cohort was predominantly female (71.5%), with a median age at symptom onset of 32.2 years (IQR 25.8–40.3) and a median age at diagnosis of 32.8 years (IQR 26.3–41.5). Median baseline EDSS score was 2.5 (IQR 1.5–3.5), while the median ARR before HET initiation was 0.24 (IQR 0.00–0.71). The median interval from symptom onset to HET initiation was 2.5 years (IQR 0.7–6.3), and 45.1% of patients started HET within two years of disease onset. The distribution of treatment timing was broad, with 256 patients (54.9%) initiating HET more than two years after symptom onset and 150 patients (32.2%) initiating HET more than five years after symptom onset. Natalizumab was the most frequently used HET (26.0%), followed by fingolimod (20.8%), ocrelizumab (20.2%), and cladribine (13.3%).

**Table 1:** Baseline characteristics of patients initiating a first high-efficacy therapy.

Characteristic	Overall cohort (N = 466)
Female sex, n (%)	333 (71.5)
Age at onset, years, median (IQR)	32.2 (25.8–40.3)
Age at diagnosis, years, median (IQR)	32.8 (26.3–41.5)
Baseline EDSS, median (IQR)	2.5 (1.5–3.5)
ARR before HET, median (IQR)	0.24 (0.00–0.71)
Time from onset to HET, years, median (IQR)	2.51 (0.68–6.30)
Time from diagnosis to HET, years, median (IQR)	1.92 (0.28–5.37)
Onset phenotype: Myelitis, n (%)	162 (34.8)
Onset phenotype: Optic pathway involvement, n (%)	117 (25.1)
Onset phenotype: Supratentorial involvement, n (%)	107 (23.0)
Onset phenotype: Brainstem involvement, n (%)	97 (20.8)
HET initiated ≤2 years from onset, n (%)	210 (45.1)
HET initiated >2 years from onset, n (%)	256 (54.9)
HET initiated ≤5 years from onset, n (%)	316 (67.8)
HET initiated >5 years from onset, n (%)	150 (32.2)
HET initiated, n (%)	
Natalizumab	121 (26.0)
Fingolimod	97 (20.8)
Ocrelizumab	94 (20.2)
Cladribine	62 (13.3)
Ofatumumab	35 (7.5)
Ozanimod	14 (3.0)
Alemtuzumab	12 (2.6)
Rituximab	12 (2.6)
Mitoxantrone	10 (2.1)
Siponimod	6 (1.3)
Ponesimod	2 (0.4)
Cyclophosphamide	1 (0.2)
Neuroimaging information	
Brain MRI available (± 12 months from baseline), n/N (%)	453/466 (97.2)
≥10 brain T2 lesions, n/N available (%)	285/435 (65.5)
Gadolinium-enhancing lesion status available, n/N (%)	277/453 (61.1)
≥1 gadolinium-enhancing brain lesion, n/N available (%)	148/277 (53.4)
Brain MRI-to-baseline interval, months, median (IQR)	0.03 (-0.56–0.39)
Follow-up information	
Visits per year before HET, median (IQR)	2.55 (1.71–4.87)
Visits per year after HET, median (IQR)	1.97 (1.51-2.27)
Post-HET follow-up, years, median (IQR)	4.05 (2.34–6.88)
Developed PIRA after HET, n (%)	70 (15.0)

ARR, annualized relapse rate; EDSS, Expanded Disability Status Scale; HET, high-efficacy therapy; IQR, interquartile range; MRI, magnetic resonance imaging; PIRA, progression independent of relapse activity; T2, T2-weighted MRI lesions.

Baseline brain MRI within ±12 months from clinical baseline was available for 453 patients (97.2%). Among patients with available baseline brain MRI, T2 lesion burden was available for 435 patients and gadolinium-enhancing lesion status for 277 patients. Overall, 285 of 435 patients (65.5%) had ≥10 brain T2 lesions, and 148 of 277 patients (53.4%) had at least one gadolinium-enhancing lesion. Brain MRI examinations were performed very close to the clinical baseline visit, with a median interval of 0.03 months (IQR −0.56 to 0.39). The median interval between baseline brain MRI and first HET initiation was 2.3 years (IQR 0.61 to 6.22).

### Predictors of PIRA occurring after first HET initiation

**Figure 2:**
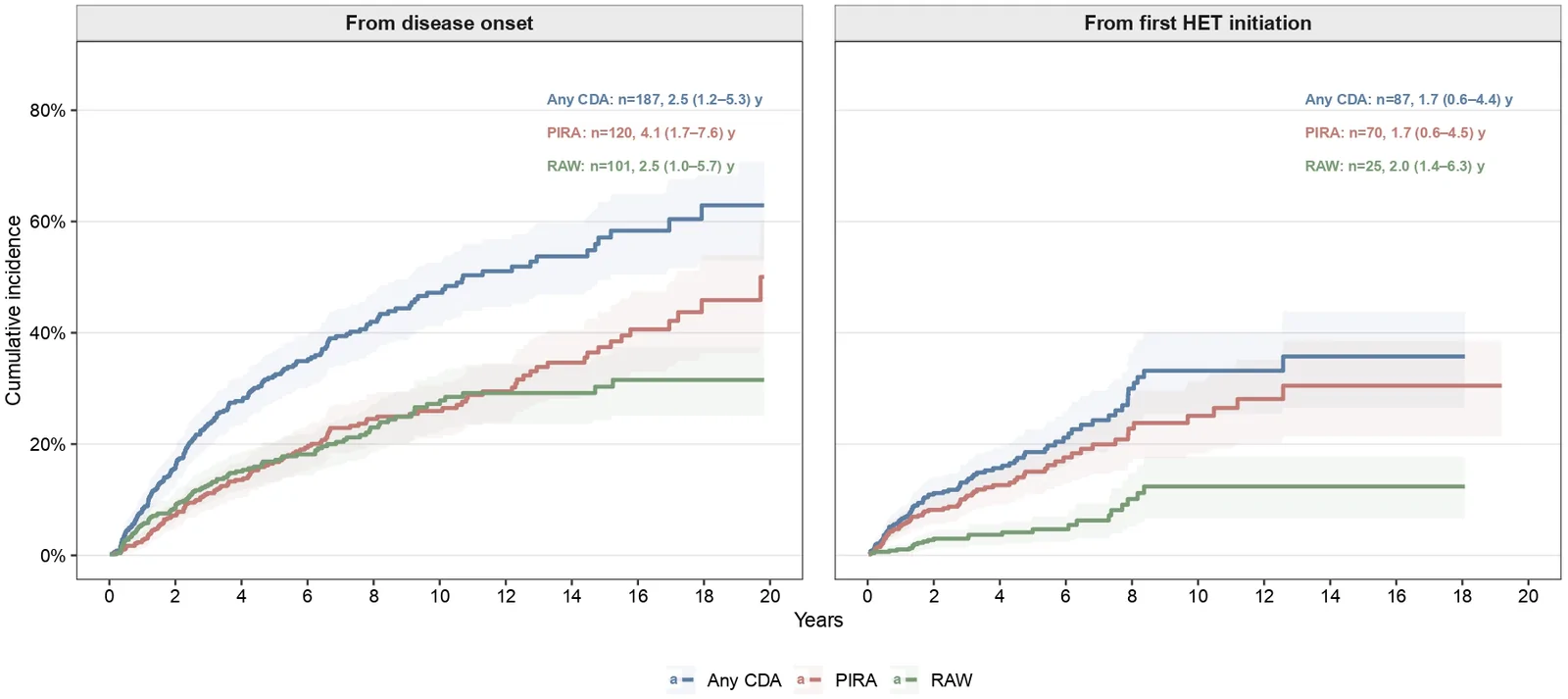
Cumulative incidence of first PIRA, RAW, and any CDA from disease onset and from HET initiation. Curves represent cumulative incidence-like estimates calculated as 1 minus Kaplan–Meier survival probability for the first occurrence of progression independent of relapse activity (PIRA), relapse-associated worsening (RAW), and any confirmed disability accrual (CDA). The left panel uses disease onset as the time origin, whereas the right panel uses initiation of the first high-efficacy therapy (HET). Labels report the number of first events and the observed median time to first event with interquartile range among patients who experienced the corresponding event. Follow-up was truncated at 20 years. Shaded areas indicate 95% confidence intervals. Curves describe time to the first event and do not account for recurrent disability worsening events. CDA, confirmed disability accrual; HET, high-efficacy therapy; PIRA, progression independent of relapse activity; RAW, relapse-associated worsening.

Overall, 70 patients (15.0%) experienced PIRA after first HET initiation. The median time to first PIRA event was 4.1 years (IQR 1.7–7.6) from symptom onset and 1.7 years (IQR 0.6–4.5) from HET initiation (**Figure 2**). Compared with patients who remained free from PIRA, those developing PIRA were older at disease onset (37.5 vs 31.5 years; p<0.001) and initiated HET later in the disease course (3.7 vs 2.4 years from symptom onset; p=0.004), whereas baseline disability, relapse activity, onset phenotype, and MRI characteristics were broadly similar between groups (**Supplementary Table 1**).

**Figure 3:**
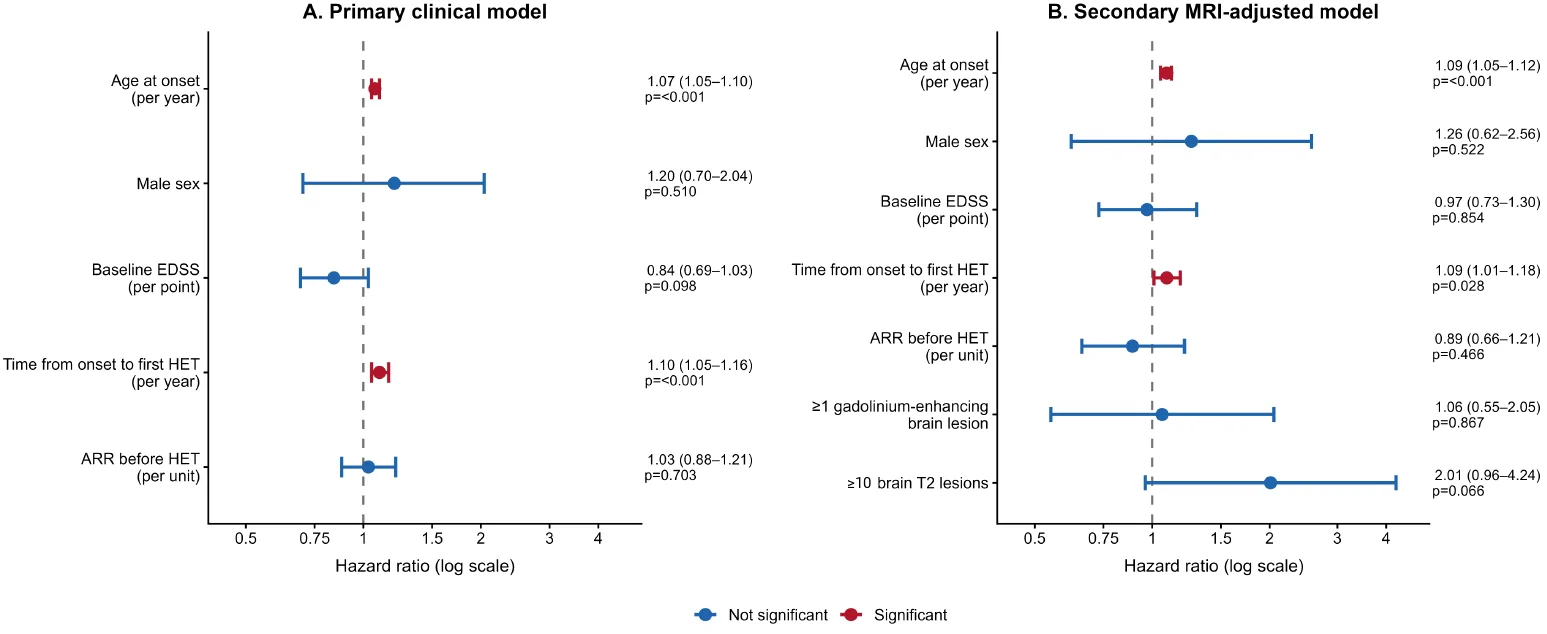
Clinical and brain MRI predictors of progression independent of relapse activity (PIRA) after first exposure to high-efficacy therapy. Forest plots showing hazard ratios (HRs) and 95% confidence intervals (CIs) for predictors of progression independent of relapse activity (PIRA) after initiation of the first high-efficacy therapy (HET). Panel **(A)** shows the primary multivariable model including prespecified clinical variables only. Panel **(B)** shows the secondary model additionally adjusted for baseline brain MRI characteristics, including the presence of gadolinium-enhancing lesions and high T2 lesion burden (≥10 lesions). Red symbols indicate statistically significant associations (p<0.05), whereas blue symbols indicate non-significant associations. The vertical dashed line represents HR = 1 (no association). Hazard ratios >1 indicate an increased risk of subsequent PIRA. ARR, annualized relapse rate; CI, confidence interval; EDSS, Expanded Disability Status Scale; HET, high-efficacy therapy; HR, hazard ratio; MRI, magnetic resonance imaging; PIRA, progression independent of relapse activity.

In univariable analyses, older age at onset, older age at diagnosis, and longer intervals from symptom onset or diagnosis to HET initiation were associated with a higher risk of subsequent PIRA (**Supplementary Table 2**). In the primary multivariable model, both older age at onset (HR 1.07, 95% CI 1.05–1.10; p<0.001) and longer time from symptom onset to HET initiation (HR 1.10, 95% CI 1.05–1.16; p<0.001) were independently associated with the occurrence of PIRA. Complete data for all clinical covariates and prespecified brain MRI variables required for the MRI-adjusted model were available for 269 patients (57.7%). Patients included in the MRI-adjusted model and those excluded because of incomplete MRI-adjusted model data did not differ significantly in key demographic and clinical characteristics (**Supplementary Table 3**). In this subset, the associations of age at onset and time to HET initiation with PIRA remained consistent after adjustment for baseline brain MRI characteristics (**Figure 3**). Among MRI variables, greater baseline brain T2 lesion burden showed only a borderline association with PIRA risk (HR 2.01, 95% CI 0.96–4.24; p=0.066).

The primary clinical model, evaluated in the full cohort, showed good discrimination for PIRA risk, with a Harrell’s C-index of 0.760 (95% CI 0.687–0.834). In the complete-case subset used for the MRI-adjusted analysis, the corresponding model had a C-index of 0.782 (95% CI 0.713–0.851) (**Supplementary Figure 1**). Because these estimates were obtained from different analytical samples, they were not directly compared.

### Exploratory analyses

#### Combined effect of age at onset and timing of HET initiation on PIRA risk

**Figure 4:**
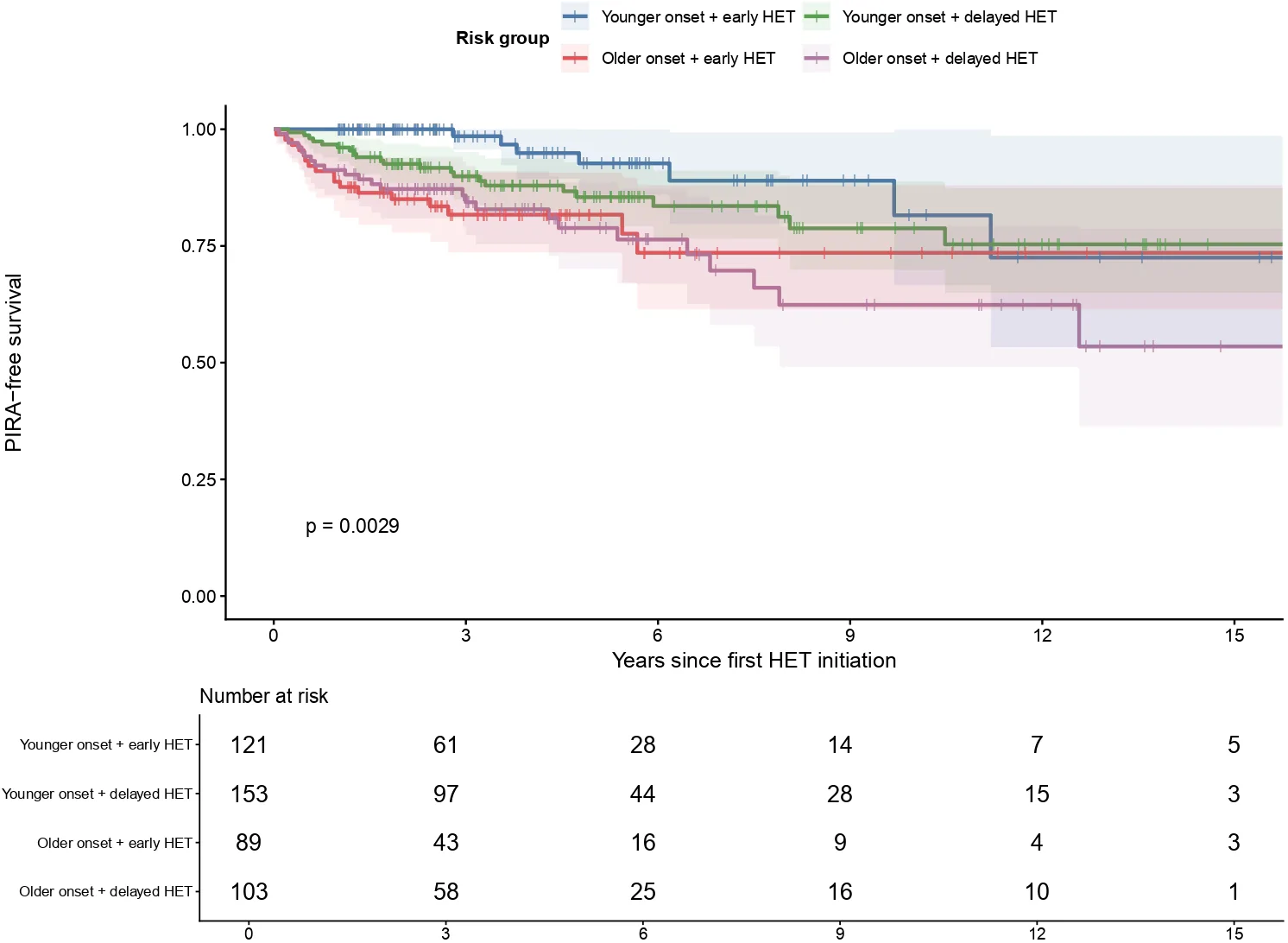
PIRA-free survival according to combined age-at-onset and timing-of-HET initiation risk strata. Kaplan–Meier estimates of PIRA-free survival following first high-efficacy therapy (HET) initiation according to combined risk groups defined by age at disease onset (<35 vs ≥35 years) and timing of HET initiation (≤2 vs >2 years from symptom onset). Shaded areas represent 95% confidence intervals. The number-at-risk table is displayed below the plot. Patients with older disease onset exhibited a higher risk of subsequent PIRA regardless of treatment timing, whereas younger-onset patients initiating HET within 2 years showed the most favorable long-term PIRA-free survival. CI, confidence interval; HET, high-efficacy therapy; KM, Kaplan–Meier; PIRA, progression independent of relapse activity.

Age at onset and timing of HET initiation showed a combined effect on PIRA risk (**Figure 4**). Patients with younger onset who received HET within two years from symptom onset had the most favorable prognosis, whereas older-onset patients showed a markedly higher risk of PIRA regardless of treatment timing. Compared with the younger onset/early HET group, adjusted HRs were 7.66 (95% CI 2.17–27.0; p=0.002) and 6.23 (95% CI 1.75–22.2; p=0.005) for older-onset patients receiving early and delayed HET, respectively. Delayed HET initiation was not independently associated with PIRA among younger-onset patients (HR 2.80, 95% CI 0.72–10.9; p=0.136). Restricted cubic spline analyses confirmed a progressive increase in PIRA risk with increasing age at onset and longer delays to HET initiation, without evidence of a clear threshold effect (**Supplementary Figure 2**).

### HET strategy-specific analysis

Among the 466 patients included in the study, 183 (39.3%) initiated HET as first-line therapy and 283 (60.7%) after previous disease-modifying treatment exposure. PIRA occurred in 26 (14.2%) and 44 (15.5%) patients, respectively. As shown in **Supplementary Figure 3**, older age at onset was consistently associated with PIRA across treatment strategies. Longer time from symptom onset to HET initiation was associated with PIRA only among switcher/escalation patients (HR 1.12, 95% CI 1.01–1.24; p=0.031), although formal interaction testing did not support effect modification by treatment strategy (p-interaction=0.347). No other clinical or brain MRI variables were independently associated with PIRA.

### Event-context analysis of predictors of disability worsening

**Figure 5:**
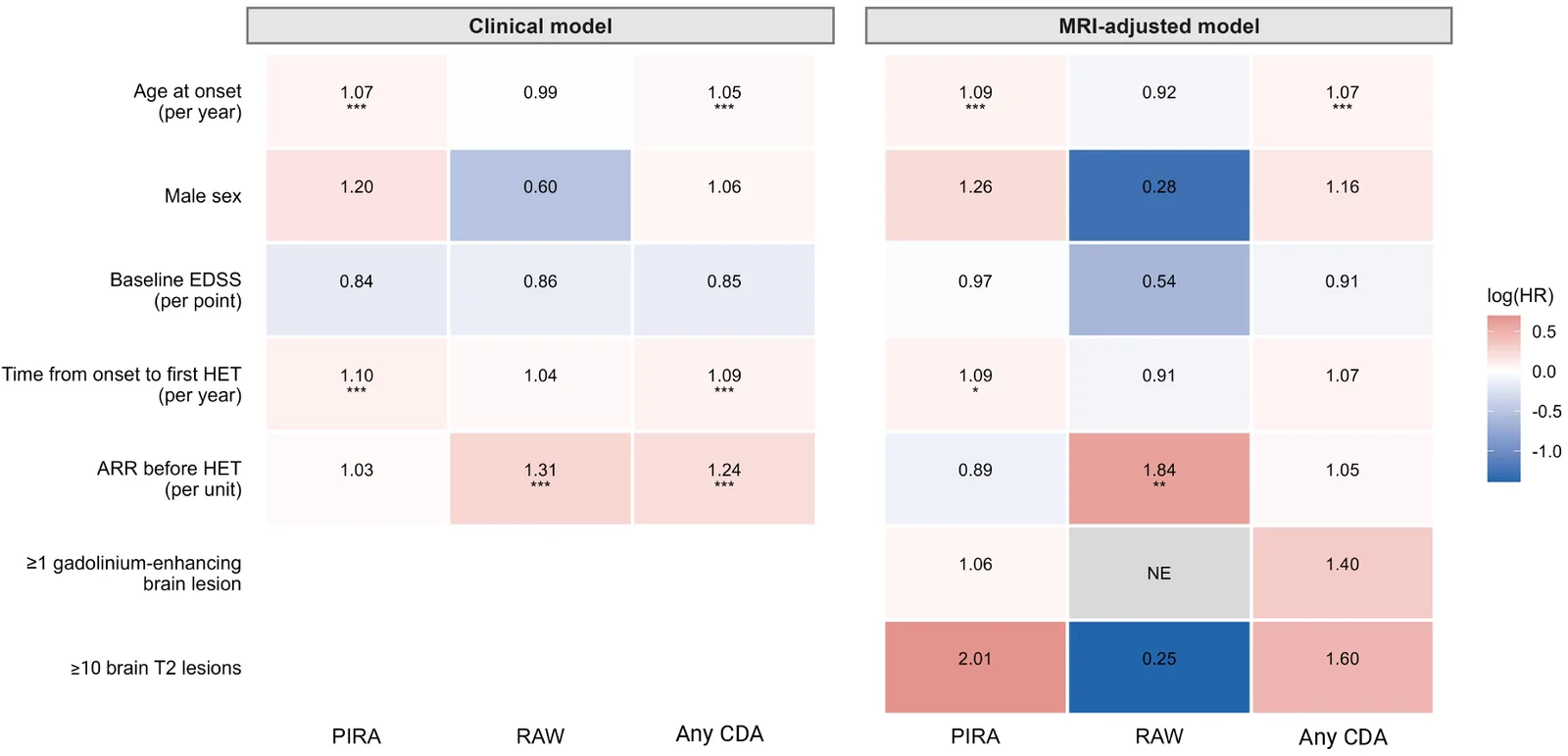
Event-context analysis of predictors of disability accumulation after HET exposure. Heatmap summarizing hazard ratios (HRs) from multivariable Cox regression models evaluating predictors of progression independent of relapse activity (PIRA), relapse-associated worsening (RAW), and any confirmed disability accumulation (CDA) after high-efficacy therapy (HET) exposure. The left panel shows the primary clinical model including age at onset, sex, baseline Expanded Disability Status Scale (EDSS) score, time from symptom onset to HET initiation, and annualized relapse rate (ARR) before HET exposure. The right panel shows the model additionally adjusted for baseline brain MRI characteristics, including gadolinium-enhancing lesions and high T2 lesion burden (≥10 lesions). Cell color intensity reflects the magnitude and direction of the association (log-transformed HR), with red indicating increased risk and blue indicating decreased risk. Numerical values represent HR estimates. Asterisks denote statistical significance (*p<0.05, **p<0.01, ***p<0.001). “NE” indicates coefficients that could not be reliably estimated because of sparse events and quasi-complete separation. ARR, annualized relapse rate; CDA, confirmed disability accumulation; EDSS, Expanded Disability Status Scale; Gd+, gadolinium-enhancing; HET, high-efficacy therapy; HR, hazard ratio; MRI, magnetic resonance imaging; NE, not estimable; PIRA, progression independent of relapse activity; RAW, relapse-associated worsening.

In an exploratory event-context analysis (**Supplementary Table 4**; **Figure 5**), predictors differed according to the mechanism of disability worsening. Among 466 patients included in the clinical model, 70 developed PIRA, 25 RAW, and 87 any CDA. Older age at onset and longer time from symptom onset to HET initiation were independently associated with PIRA (HR 1.07 and 1.10, respectively; both p<0.001), but not with RAW. Conversely, higher pre-HET relapse activity was associated with RAW (HR 1.31, 95% CI 1.18–1.46; p<0.001) but not with PIRA. Similar findings were observed in MRI-adjusted models (n=269), in which age at onset and treatment delay remained associated with PIRA, whereas pre-HET relapse activity remained associated with RAW.

### PIRA risk according to first HET class

In an exploratory analysis according to first HET class, cumulative PIRA incidence differed across treatment groups (log-rank p=0.043), with patients receiving anti-CD20 therapies showing the highest unadjusted cumulative incidence of PIRA over follow-up (**Supplementary Figure 4**). However, after adjustment for clinical characteristics, no HET class was independently associated with PIRA risk compared with anti-CD20 therapies. Similar findings were observed in MRI-adjusted models, although induction/immune reconstitution therapies [predominantly cladribine (n=62) and alemtuzumab (n=12)] were associated with a lower risk of PIRA than anti-CD20 therapies (HR 0.31, 95% CI 0.10–0.97; p=0.044) (**Supplementary Figure 5**).

### Sensitivity analyses

Results were consistent in an on-treatment sensitivity analysis censoring follow-up at treatment discontinuation or treatment switch, with age at onset and time to HET initiation remaining independently associated with PIRA (**Supplementary Figure 6**). Findings were similarly unchanged in a 90-day landmark analysis excluding early PIRA events. Six of 70 patients with PIRA (8.6%) experienced the event within 90 days of HET initiation and were therefore excluded. In both the clinical and MRI-adjusted models, older age at onset and longer time from symptom onset to HET initiation remained independently associated with subsequent PIRA (**Supplementary Figure 7**). In the EDSS re-baselining sensitivity analysis, an eligible assessment was available for 348 patients (74.7%). Despite some PIRA event reclassification, older age at onset and longer time to HET initiation remained independently associated with PIRA, whereas EDSS at HET initiation was not; findings were consistent in the MRI-adjusted model (**Supplementary Figure 8**). Finally, further adjustment for calendar year of first HET initiation did not materially alter the main associations (**Supplementary Figure 9**).

## Discussion

In this multicenter real-world study of patients with relapsing MS treated with a first HET, older age at disease onset and longer time from symptom onset to first HET initiation emerged as the strongest and most consistent predictors of subsequent PIRA, independently of baseline disability, relapse activity, and MRI characteristics. Predictors of PIRA also differed from those of RAW, suggesting partially distinct, although potentially overlapping, pathways of disability accumulation.

Age at disease onset was the most robust predictor of PIRA, consistent with evidence that progression risk increases with advancing age (11, 16), including during the relapsing phase of MS (5). Potential mechanisms include immunosenescence, reduced remyelination capacity and neurological reserve, and accumulation of compartmentalized inflammatory and neurodegenerative processes (17). Age may therefore represent a major determinant of progression susceptibility that is only partially modifiable by current anti-inflammatory therapies (16). This interpretation is supported by large early-MS cohorts. In the Italian Multiple Sclerosis Register study by Portaccio et al., clinically defined PIRA occurred in 27.6% of 5,169 patients over a mean follow-up of 11.5 years, and older age was independently associated with both PIRA and true PIRA (4). Similarly, in the Barcelona first-demyelinating-event cohort by Tur et al., older age at first attack was the only baseline predictor of subsequent PIRA (6).

Longer time from symptom onset to HET initiation was also associated with higher PIRA risk, in keeping with the concept of an early therapeutic window in MS (18). However, this association should be interpreted cautiously. Treatment timing was not randomized and likely reflected diagnostic delay, evolving access to HETs, perceived disease severity, physician and patient preferences, and changes in therapeutic strategies. This is particularly relevant for patients whose disease began before HETs became widely available, in whom a longer interval may reflect historical treatment unavailability rather than a modifiable delay. Time to HET should therefore be regarded as a clinically informative risk marker rather than as a causal estimate of the effect of delayed treatment. Nevertheless, the association remained consistent across multivariable, MRI-adjusted, spline, on-treatment, landmark, EDSS re-baselining, and calendar-year-adjusted analyses. Although re-baselining EDSS at HET initiation led to some PIRA event reclassification, the associations of age at onset and time to HET remained essentially unchanged.

Age and treatment timing appeared to contribute independently to progression risk. Patients with younger onset who initiated HET within two years had the most favorable prognosis, whereas older-onset patients had substantially higher PIRA risk regardless of treatment timing. Thus, although early treatment may reduce risk, a relevant component remains associated with biological aging.

Routine baseline conventional brain MRI lesion metrics showed limited independent prognostic value in the complete-case MRI-adjusted analysis. However, baseline lesion burden differs conceptually from new or enlarging T2 lesions and gadolinium-enhancing lesions occurring around disability-worsening events. In the Barcelona cohort, recent MRI activity was present in 73 of 144 (51%) evaluable PIRA cases. Similarly, in the MRI-evaluable subgroup of the Italian Multiple Sclerosis Register, 172 events were classified as PIRA clinically, but only 84 fulfilled the true-PIRA definition after MRI activity was considered (6). Thus, clinically defined PIRA events were reduced by approximately half when recent radiological activity was excluded. Clinically defined PIRA therefore cannot be equated with purely neurodegenerative progression and may include disability accumulation associated with subclinical focal inflammation. This distinction is biologically plausible because acute inflammatory lesions can cause irreversible axonal injury. Histopathological examination of active MS lesions demonstrated an average of 11,236 transected axons per cubic millimeter, directly linking focal inflammation with permanent tissue damage (19). Consequently, the association between earlier HET initiation and lower clinically defined PIRA risk may partly reflect more effective suppression of acute or subclinical MRI activity, alongside effects on compartmentalized inflammation, neurodegeneration, and neurological reserve. Adjustment for baseline MRI characteristics cannot distinguish these mechanisms because MRI activity proximal to each PIRA event was unavailable.

Findings from the French Multiple Sclerosis Registry further emphasize the distinction between clinically defined PIRA and progression independent of relapse and MRI activity (PIRMA or “true PIRA”). Among 10,499 patients with relapsing-onset MS, Rollot et al. found that first-line HET provided better control of relapse-associated and MRI-associated worsening than moderate-efficacy therapies, but did not prolong time to PIRMA. Restricted mean time to PIRMA was similar and slightly shorter in the HET group, whereas older age, higher baseline EDSS, and spinal cord lesions were associated with PIRMA (20). These findings suggest that part of the apparent effect of HET on clinically defined PIRA may be mediated through suppression of focal inflammatory activity, whereas progression occurring independently of both relapses and MRI activity may be less responsive to currently available anti-inflammatory therapies. Our results should therefore be interpreted as identifying predictors of clinically defined PIRA, not as demonstrating exclusively neurodegenerative mechanisms.

The distinct clinical predictor profiles observed for PIRA and RAW provide a further relevant finding. Older age at onset and longer time to HET were associated with clinically defined PIRA, whereas higher pre-HET relapse activity predicted RAW but not PIRA. This divergence supports partially distinct clinical patterns but does not demonstrate the absence of inflammatory mechanisms in PIRA. Relapses and MRI lesion activity represent different manifestations of acute focal inflammation, and event-proximal subclinical MRI activity was not available in our cohort. Earlier HET initiation may therefore reduce PIRA risk partly through suppression of subclinical lesion activity, alongside age-related vulnerability, reduced neurological reserve, chronic compartmentalized inflammation, and neurodegenerative processes (21, 22). Clinically defined PIRA and RAW should consequently be considered overlapping pathways of disability accrual rather than direct proxies for purely neurodegenerative and purely inflammatory mechanisms.

### Strengths and limitations

This study leveraged two prospectively maintained MS registries with standardized clinical, disability, treatment, and MRI data, enabling detailed longitudinal characterization. Robustness was supported by complementary MRI-adjusted, strategy-specific, spline, risk-stratification, event-context, on-treatment, landmark, EDSS re-baselining, and calendar-year-adjusted analyses. Several limitations should be acknowledged. First, residual confounding, including confounding by indication, cannot be excluded. Time from symptom onset to HET initiation was not randomized and may partly reflect calendar-time changes in HET availability, access, and therapeutic practice, together with diagnostic delay, disease severity, clinical decision-making, and patient-related factors. Although adjustment for calendar year of first HET initiation did not materially alter the association, calendar year is only a partial proxy for these evolving contextual factors. Time to HET should therefore be interpreted as a risk marker rather than a causal estimate of treatment delay. Second, MRI-adjusted and exploratory subgroup analyses included fewer events, resulting in wider confidence intervals and reduced power, particularly for MRI variables and interactions. Disability worsening was defined through EDSS-based confirmed disability accumulation, which may incompletely capture cognitive impairment, upper-limb dysfunction, fatigue, and other subtle manifestations of progression. Moreover, PIRA was defined by the absence of relapses within the prespecified temporal window and did not require the absence of new or enlarging T2 lesions or gadolinium-enhancing lesions around disability worsening. Previous event-centered MRI studies indicate that approximately half of clinically defined PIRA events may occur with recent MRI activity. Baseline MRI adjustment does not resolve this limitation because baseline lesion burden differs from new inflammatory activity proximal to an individual event. Some events classified as PIRA may therefore have been associated with subclinical focal inflammation, and our findings apply to clinically defined PIRA rather than PIRMA or true PIRA. PIRA and RAW were also analyzed as outcome-specific events rather than formal competing risks and may overlap within individual patients. Third, complete-case requirements reduced the MRI-adjusted sample to 269 patients. Baseline MRI was anchored to the early clinical baseline and was not necessarily performed immediately before HET initiation, potentially weakening its ability to predict post-HET PIRA in patients with longer pre-HET disease duration. MRI analyses were also limited to routine conventional brain lesion measures and did not include spinal lesions or quantitative and advanced markers of neurodegeneration, chronic inflammatory activity, and diffuse tissue injury. Finally, treatment allocation reflected real-world practice over more than two decades, during which treatment availability, monitoring intensity, and therapeutic strategies changed substantially. Unmeasured differences in disease severity, adherence, access to care, and clinical decision-making may have influenced both HET initiation and outcomes. Although multicenter, the cohort was derived from two Italian centers and predominantly included White European patients, potentially limiting generalizability to other populations and healthcare settings.

In conclusion, older age at disease onset and longer time to HET initiation were the strongest and most consistent predictors of clinically defined PIRA after HET exposure, and their associations were robust across multiple complementary analyses. However, these findings should not be interpreted as demonstrating that PIRA is purely neurodegenerative or independent of subclinical MRI inflammatory activity. Future studies incorporating standardized brain and spinal MRI assessments proximal to disability-worsening events are needed to determine whether these predictors also apply to PIRMA or true PIRA.

## Data Availability

The raw data supporting the conclusions of this article will be made available by the authors, without undue reservation.
