# Supplementary material for: Predictors of subsequent relapse-independent progression after high-efficacy therapy in relapsing multiple sclerosis
Source: Front Immunol. 2026 Aug 19;17:1935407. doi: 10.3389/fimmu.2026.1935407 (PMC13533764; doi:10.3389/fimmu.2026.1935407)
Supplement: Supplementary file 1 [file DataSheet1.pdf]

Supplementary Material

Predictors of Subsequent Relapse-Independent Progression After High-Efficacy Therapy in Relapsing Multiple Sclerosis

Matteo Foschi, MD, PhD,[1*] Andrea Surcinelli, MD,[1] Ivan Panzera, MD,[1] Ludovica Maria Miserocchi, MD,[1] Giorgia Balducci, MD,[1] Luca Mancinelli, MD,[2] Cristiana Ganino, MD,[3] Francesca Graziano,[4,5] Ester Lagonigro, MD,[6] Maria Di Ioia, MD, PhD,[7] Valeria Di Tommaso, MD, PhD,[7] Deborah Farina, MD,[7] Giovanna De Luca, MD,[7] Massimo Caulo, MD, PhD,[4] Maria Grazia Piscaglia, MD,[1] Stefano L. Sensi, MD, PhD,[4,6,7] Valentina Tomassini, MD, PhD[4,5,6,7*]

1) Department of Neurosciences, Multiple Sclerosis Center - Neurology Unit, S. Maria delle Croci Hospital, AUSL Romagna, Ravenna, Italy.
2) Department of Neurosciences, Multiple Sclerosis Center - Neurology Unit, Maurizio Bufalini Hospital, AUSL Romagna, Cesena, Italy.
3) Department of Neurosciences, Multiple Sclerosis Center - Neurology Unit, Infermi Hospital, AUSL Romagna, Rimini, Italy.
4) Institute of Advanced Biomedical Technologies (ITAB), Department of Neurosciences, Imaging and Clinical Sciences, University G. d'Annunzio of Chieti-Pescara, Chieti, Italy.
5) Center for Disability, Rehabilitation and Sports Medicine (CARES), Department of Neuroscience and Clinical Sciences, University of Chieti, Chieti, Italy.
6) Department of Neurosciences, Imaging and Clinical Sciences, University G. d'Annunzio of Chieti-Pescara, Chieti, Italy.
7) MS Centre, Neurology Institute, SS. Annunziata University Hospital, Chieti, Italy.

Corresponding authors:

- Matteo Foschi, MD, PhD; Department of Neurosciences, Multiple Sclerosis Center – Neurology Unit, S. Maria delle Croci Hospital, AUSL Romagna, Ravenna, Italy. Viale Vincenzo Randi 5, 48121, Ravenna, Italy. E-mail: matteo.foschi@auslromagna.it; orcid.org/0000-0002-0321-7155.

- Valentina Tomassini, MD, PhD; Department of Neurosciences, Imaging and Clinical Sciences, University G. D'Annunzio, Chieti-Pescara, Italy; Via dei Vestini 33, 66100, Chieti, Italy. E-mail: valentina.tomassini@unich.it; orcid.org/0000-0002-7368-6280.

Supplementary Tables

Supplementary Table 1. Baseline characteristics according to development of PIRA after HET exposure.

Supplementary Table 2. Univariable Cox regression analyses for the risk of PIRA after HET exposure.

Supplementary Table 3. Key characteristics of patients included in and excluded from the MRI-adjusted model.

Supplementary Table 4. Predictors of PIRA, RAW, and any CDA after HET exposure.

Supplementary Figures

Supplementary Figure 1. Discriminative performance of clinical and MRI-adjusted models for predicting PIRA after HET exposure.

Supplementary Figure 2. Continuous associations of age at onset and time to HET initiation with the risk of PIRA.

Supplementary Figure 3. Strategy-specific clinical and brain MRI predictors of PIRA after exposure to high-efficacy therapy.

Supplementary Figure 4. Cumulative incidence of PIRA according to HET class.

Supplementary Figure 5. Association between HET class and risk of PIRA: clinical and MRI-adjusted Cox models.

Supplementary Figure 6. On-treatment sensitivity analysis of clinical and MRI predictors of PIRA after HET exposure.

Supplementary Figure 7. Landmark analysis with a 90-day lag period: clinical and MRI predictors of PIRA after HET exposure.

Supplementary Figure 8. Sensitivity analysis using EDSS re-baselined at first high-efficacy therapy initiation.

Supplementary Figure 9. Sensitivity analysis adjusted for calendar year of first high-efficacy therapy initiation.

Supplementary Tables

Supplementary Table 1. Baseline characteristics according to development of PIRA after HET exposure.

Characteristic	No PIRA after HET (n=396)	PIRA after HET (n=70)	p-value
Female sex, n (%)	282 (71.2)	51 (72.9)	0.779
Age at onset, years	31.5 (25.5–39.5)	37.5 (27.7–47.6)	<0.001
Age at diagnosis, years	32.1 (25.8–40.1)	38.3 (30.0–47.9)	<0.001
Baseline EDSS	2.5 (1.5–3.5)	2.0 (1.0–3.5)	0.276
Time from onset to HET, years	2.4 (0.6–5.8)	3.7 (1.2–9.0)	0.004
Time from diagnosis to HET, years	1.7 (0.3–5.1)	2.9 (0.8–8.2)	0.016
ARR before HET	0.22 (0.00–0.72)	0.34 (0.00–0.61)	0.385
Myelitis at onset, n (%)	137 (34.6)	25 (35.7)	0.856
Brainstem involvement at onset, n (%)	83 (21.0)	14 (20.0)	0.855
Optic pathway involvement at onset, n (%)	101 (25.5)	16 (22.9)	0.638
Supratentorial involvement at onset, n (%)	91 (23.0)	16 (22.9)	0.982
≥1 gadolinium-enhancing brain lesion, n/N available (%)	126 (52.9)	22 (56.4)	0.687
≥10 brain T2 lesions, n/N available (%)	243 (66.0)	42 (62.7)	0.596

Data are presented as median (interquartile range) or n (%). P-values were calculated using the Wilcoxon rank-sum test for continuous variables and Pearson's χ^2 test or Fisher's exact test, as appropriate. Percentages for MRI lesion variables were calculated among patients with available MRI data. ARR = annualized relapse rate; EDSS = Expanded Disability Status Scale; HET = high-efficacy therapy; MRI = magnetic resonance imaging; PIRA = progression independent of relapse activity.

Supplementary Table 2. Univariable Cox regression analyses for the risk of PIRA after HET exposure.

**Variable**	**HR (95% CI)**	**p-value**
Male sex	1.03 (0.61–1.75)	0.908
Age at onset (per year)	1.06 (1.04–1.08)	<0.001
Age at diagnosis (per year)	1.06 (1.04–1.08)	<0.001
Baseline EDSS (per point)	0.86 (0.71–1.05)	0.132
Time from onset to HET (per year)	1.09 (1.04–1.15)	<0.001
Time from diagnosis to HET (per year)	1.09 (1.04–1.15)	0.001
HET initiation >2 years from onset	1.38 (0.84–2.26)	0.204
HET initiation >5 years from onset	1.59 (0.99–2.56)	0.054
ARR before HET (per unit)	0.94 (0.78–1.13)	0.521
Myelitis at onset	0.97 (0.60–1.59)	0.918
Brainstem involvement at onset	1.00 (0.55–1.79)	0.991
Optic pathway involvement at onset	0.97 (0.55–1.70)	0.917
Supratentorial involvement at onset	0.86 (0.49–1.51)	0.596
≥1 gadolinium-enhancing brain lesion	0.96 (0.51–1.81)	0.889
≥10 brain T2 lesions	1.08 (0.66–1.78)	0.756

Hazard ratios (HRs) were estimated using univariable Cox proportional hazards models. HRs >1 indicate increased risk of PIRA after HET exposure. ARR = annualized relapse rate; EDSS = Expanded Disability Status Scale; HET = high-efficacy therapy; MRI = magnetic resonance imaging; PIRA = progression independent of relapse activity.

Supplementary Table 3. Key characteristics of patients included in and excluded from the MRI-adjusted model.

Characteristic	Included in MRI-adjusted model (n=269)	Excluded from MRI-adjusted model (n=197)	p-value
Ravenna centre, n (%)	154 (57.2)	113 (57.4)	0.960
Female sex, n (%)	193 (71.7)	140 (71.1)	0.883
Age at onset, years, median (IQR)	32.4 (25.9–40.5)	31.9 (25.6–40.0)	0.641
Baseline EDSS, median (IQR)	2.5 (1.5–3.5)	2.5 (1.5–3.5)	0.920
ARR before HET, median (IQR)	0.25 (0.00–0.71)	0.22 (0.00–0.69)	0.586
Time from onset to HET, years, median (IQR)	2.6 (0.7–6.4)	2.4 (0.6–6.1)	0.690
Switcher/escalation HET, n (%)	164 (61.0)	119 (60.4)	0.901
Post-HET follow-up, years, median (IQR)	4.1 (2.4–6.9)	3.9 (2.3–6.7)	0.622

Data are presented as median (interquartile range) or n (%). Patients included in the MRI-adjusted model had complete data for all clinical covariates and prespecified baseline brain MRI variables required for the model. P-values were calculated using the Wilcoxon rank-sum test for continuous variables and Pearson's χ^2 test or Fisher's exact test for categorical variables, as appropriate. Switcher/escalation HET refers to patients who initiated high-efficacy therapy after prior exposure to another disease-modifying therapy. Abbreviations: EDSS = Expanded Disability Status Scale; HET = high-efficacy therapy; IQR = interquartile range; MRI = magnetic resonance imaging.

Supplementary Table 4. Predictors of PIRA, RAW, and Any CDA after HET exposure.

**Clinical model**						
**Variable**	**PIRA HR (95% CI)**	**p**	**RAW HR (95% CI)**	**p**	**Any CDA HR (95% CI)**	**p**
Age at onset (per year)	1.07 (1.05–1.10)	<0.001	0.99 (0.94–1.03)	0.505	1.05 (1.03–1.08)	<0.001
Male sex	1.20 (0.70–2.04)	0.510	0.60 (0.20–1.77)	0.352	1.06 (0.65–1.74)	0.801
Baseline EDSS (per point)	0.84 (0.69–1.03)	0.098	0.86 (0.60–1.24)	0.422	0.85 (0.71–1.02)	0.085
Time from onset to HET (per year)	1.10 (1.05–1.16)	<0.001	1.04 (0.93–1.15)	0.486	1.09 (1.04–1.15)	<0.001
ARR before HET (per unit)	1.03 (0.88–1.21)	0.703	1.31 (1.18–1.46)	<0.001	1.24 (1.13–1.36)	<0.001
**Model adjusted for baseline MRI characteristics**						
**Variable**	**PIRA HR (95% CI)**	**p**	**RAW HR (95% CI)**	**p**	**Any CDA HR (95% CI)**	**p**
Age at onset (per year)	1.09 (1.05–1.12)	<0.001	0.92 (0.83–1.02)	0.130	1.07 (1.04–1.10)	<0.001
Male sex	1.26 (0.62–2.56)	0.522	0.28 (0.03–2.64)	0.266	1.16 (0.60–2.24)	0.653
Baseline EDSS (per point)	0.97 (0.73–1.30)	0.854	0.54 (0.23–1.27)	0.158	0.91 (0.69–1.20)	0.511
Time from onset to HET (per year)	1.09 (1.01–1.18)	0.028	0.91 (0.72–1.15)	0.423	1.07 (1.00–1.16)	0.063
ARR before HET (per unit)	0.89 (0.66–1.21)	0.466	1.84 (1.17–2.88)	0.008	1.05 (0.87–1.28)	0.590
≥1 Gd+ lesion	1.06 (0.55–2.05)	0.867	Not estimable*	—	1.40 (0.75–2.59)	0.286
≥10 brain T2 lesions	2.01 (0.96–4.24)	0.066	0.29 (0.25-1.31)	0.102	1.60 (0.82-3.11)	0.170

Hazard ratios (HRs) and 95% confidence intervals (CIs) from multivariable Cox proportional hazards regression models evaluating predictors of progression independent of relapse activity (PIRA), relapse-associated worsening (RAW), and any confirmed disability accumulation (CDA) after high-efficacy therapy (HET) exposure. The clinical model included age at onset, sex, baseline Expanded Disability Status Scale (EDSS) score, time from symptom onset to HET initiation, and annualized relapse rate (ARR) before HET exposure. The MRI-adjusted model additionally included the presence of gadolinium-enhancing brain lesions and high T2 lesion burden (≥10 lesions). The outcome "Any CDA" included both PIRA and RAW events. Hazard ratios >1 indicate an increased risk of the corresponding outcome. The coefficient for gadolinium-enhancing lesions in the MRI-adjusted RAW model could not be reliably estimated because of sparse events and quasi-complete separation (8 RAW events). Abbreviations: ARR, annualized relapse rate; CDA, confirmed disability accumulation; CI, confidence interval; EDSS, Expanded Disability Status Scale; Gd+, gadolinium-enhancing; HET, high-efficacy therapy; HR, hazard ratio; MRI, magnetic resonance imaging; PIRA, progression independent of relapse activity; RAW, relapse-associated worsening.

Supplementary Figures

Supplementary Figure 1. Discriminative performance of clinical and MRI-adjusted models for predicting PIRA after HET exposure.

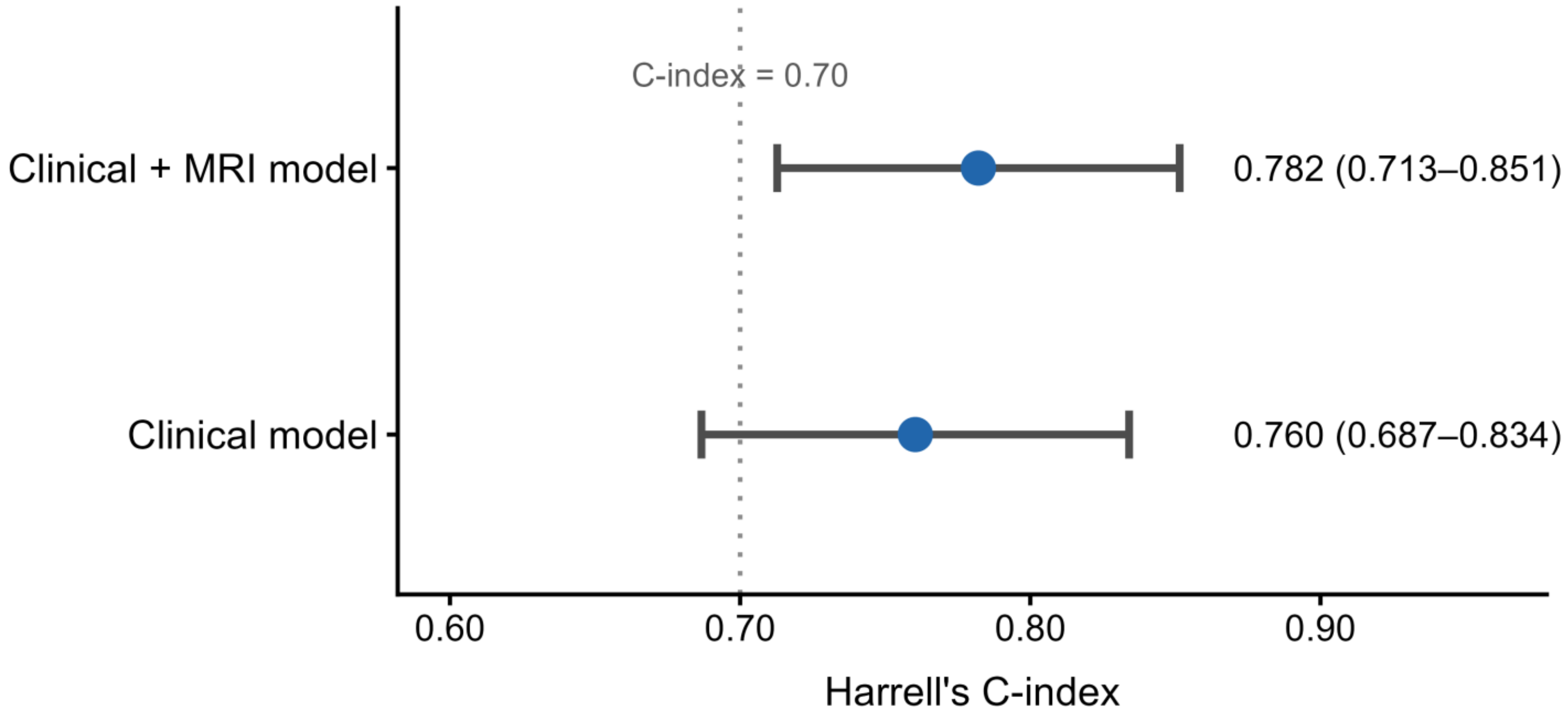

Harrell's C-index estimates and corresponding 95% confidence intervals for the primary clinical model and the secondary model additionally adjusted for baseline brain MRI characteristics. The primary clinical model included age at onset, sex, baseline EDSS, time from symptom onset to high-efficacy therapy (HET) initiation, and annualized relapse rate (ARR) before HET exposure. The MRI-adjusted model additionally included the presence of gadolinium-enhancing brain lesions and high T2 lesion burden (≥10 lesions). The clinical model was evaluated in the full cohort (n=466), whereas the MRI-adjusted model was evaluated in the complete-case MRI subset (n=269). Abbreviations: ARR, annualized relapse rate; CI, confidence interval; EDSS, Expanded Disability Status Scale; HET, high-efficacy therapy; MRI, magnetic resonance imaging; PIRA, progression independent of relapse activity.

Supplementary Figure 2. Continuous associations of age at onset and time to HET initiation with the risk of PIRA.

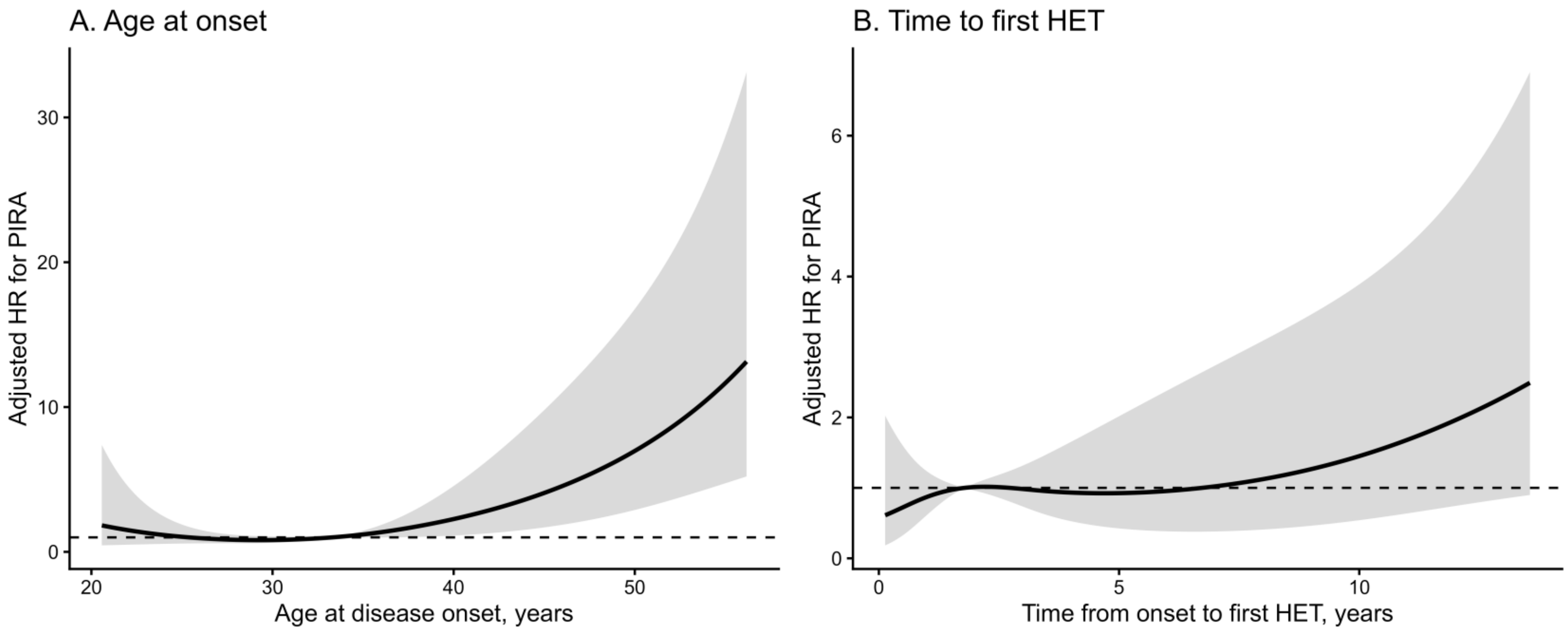

Restricted cubic spline analyses showing the adjusted hazard ratio (HR) for progression independent of relapse activity (PIRA) according to age at disease onset (Panel A) and time from symptom onset to high-efficacy therapy (HET) initiation (Panel B). Solid lines represent adjusted hazard ratios, while shaded areas indicate 95% confidence intervals. The dashed horizontal line represents the reference hazard ratio (HR=1). Models were adjusted for sex, baseline Expanded Disability Status Scale (EDSS) score, annualized relapse rate (ARR) before HET exposure, presence of gadolinium-enhancing lesions, and high brain T2 lesion burden (≥10 lesions). Risk of PIRA increased progressively with older age at onset and, to a lesser extent, with longer delays to HET initiation, without evidence of a clear threshold effect. Abbreviations: CI, confidence interval; EDSS, Expanded Disability Status Scale; HET, high-efficacy therapy; HR, hazard ratio; PIRA, progression independent of relapse activity.

Supplementary Figure 3. Strategy-specific clinical and brain MRI predictors of PIRA after exposure to high-efficacy therapy.

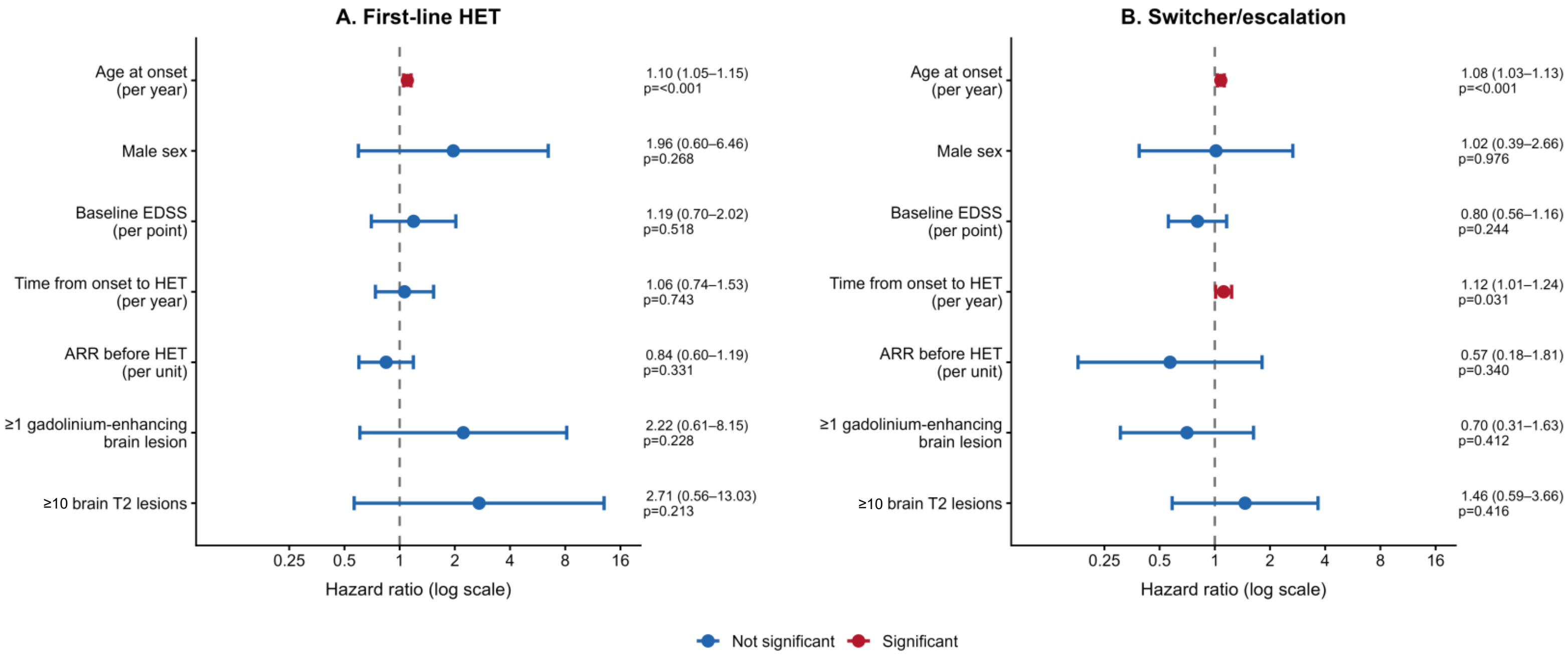

Forest plots showing hazard ratios (HRs) and 95% confidence intervals (CIs) from multivariable Cox regression models evaluating clinical and baseline brain MRI predictors of progression independent of relapse activity (PIRA) after initiation of the high-efficacy therapy (HET), stratified according to treatment strategy. Panel A includes patients who received HET as first-line therapy. Panel B includes patients who initiated HET following previous disease-modifying therapy exposure (switcher/escalation strategy). Models were adjusted for age at onset, sex, baseline EDSS, time from symptom onset to HET initiation, annualized relapse rate (ARR) before HET exposure, presence of gadolinium-enhancing brain lesions, and T2 lesion burden. Red symbols indicate statistically significant associations ($p<0.05$), whereas blue symbols indicate non-significant associations. The dashed vertical line represents HR=1 (no association). Hazard ratios >1 indicate an increased risk of subsequent PIRA. Abbreviations: ARR, annualized relapse rate; CI, confidence interval; EDSS, Expanded Disability Status Scale; HET, high-efficacy therapy; HR, hazard ratio; MRI, magnetic resonance imaging; PIRA, progression independent of relapse activity.

Supplementary Figure 4. Cumulative incidence of PIRA according to HET class.

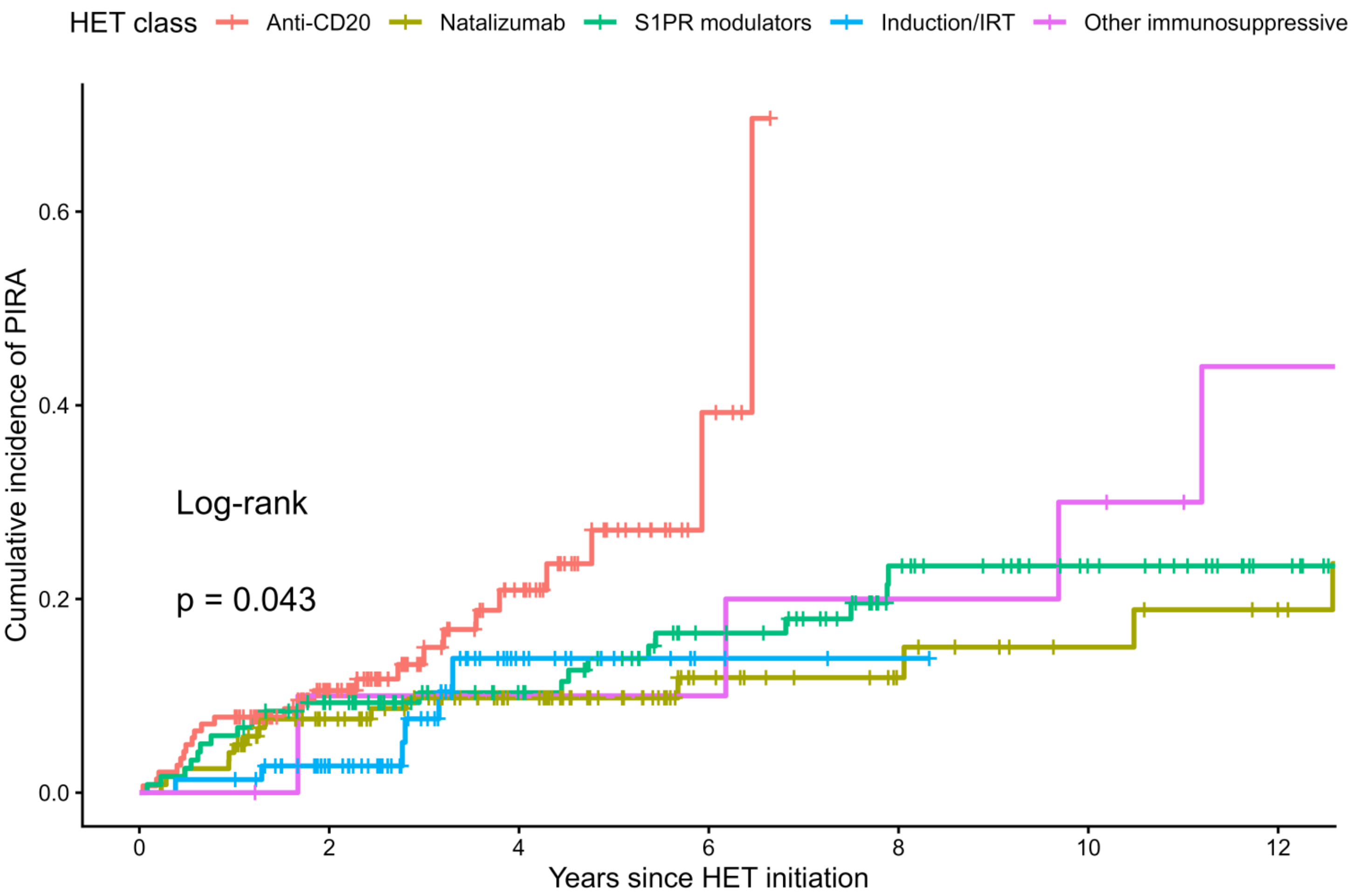

Kaplan–Meier estimates of cumulative PIRA incidence following initiation of a high-efficacy therapy (HET), stratified according to HET class. Anti-CD20 therapies included ocrelizumab, ofatumumab, and rituximab; S1PR modulators included fingolimod, ozanimod, ponesimod, and siponimod; induction/immune reconstitution therapies included cladribine and alemtuzumab; other immunosuppressive therapies included mitoxantrone and cyclophosphamide. P-values were derived from the log-rank test. Curves are unadjusted and should be interpreted descriptively. Abbreviations: PIRA, progression independent of relapse activity; HET, high-efficacy therapy; IRT, immune reconstruction therapy; S1PR, sphingosine-1-phosphate receptor.

Supplementary Figure 5. Association between HET class and risk of PIRA: clinical and MRI-adjusted Cox models.

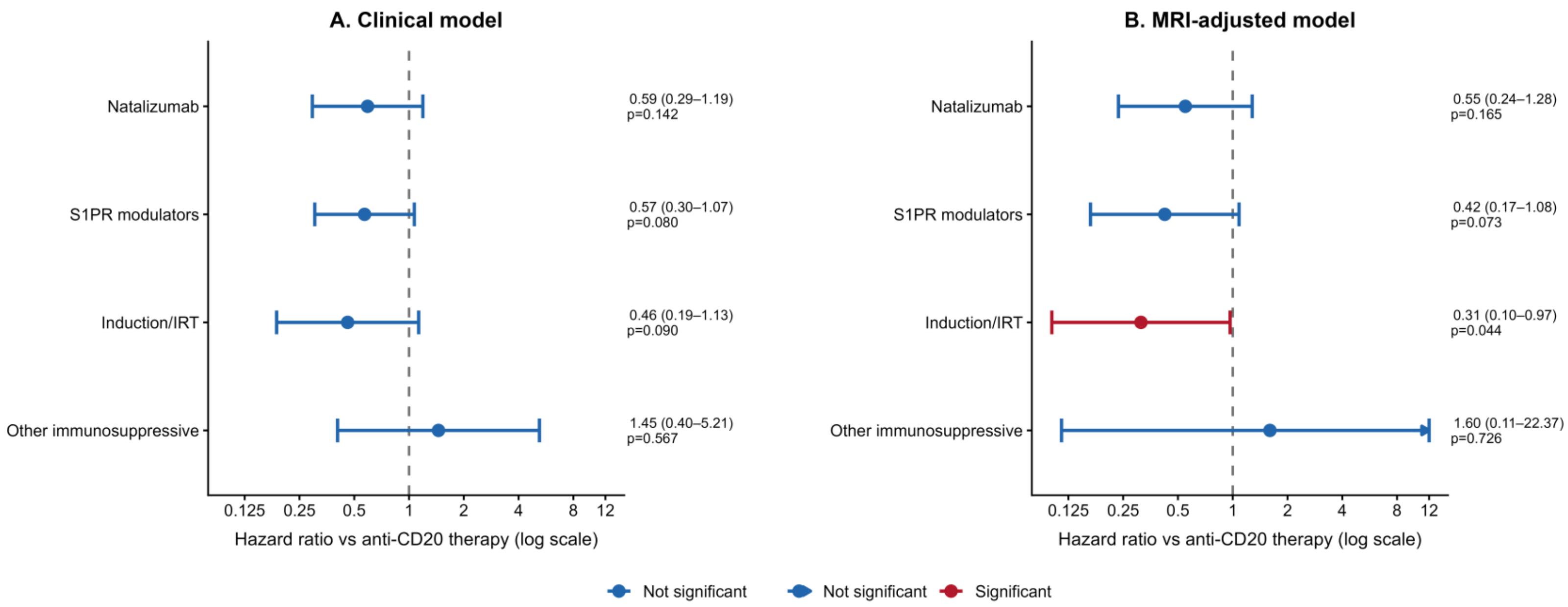

Forest plots showing adjusted hazard ratios (HRs) for PIRA according to HET class using anti-CD20 therapies as the reference category. Panel A shows the clinical model adjusted for age at onset, sex, baseline EDSS, time from symptom onset to HET initiation, and pre-HET ARR. Panel B additionally includes baseline brain MRI variables (gadolinium-enhancing lesions and high T2 lesion burden [≥10 lesions]). Induction/immune reconstitution therapies comprised cladribine and alemtuzumab. Confidence intervals extending beyond the plotting range were truncated and indicated by arrows. Abbreviations: PIRA, progression independent of relapse activity; HET, high-efficacy therapy; HR, hazard ratio; CI, confidence interval; EDSS, Expanded Disability Status Scale; ARR, annualized relapse rate; IRT, immune reconstruction therapy; MRI, magnetic resonance imaging; S1PR, sphingosine-1-phosphate receptor.

Supplementary Figure 6. On-treatment sensitivity analysis of clinical and MRI predictors of PIRA after HET exposure.

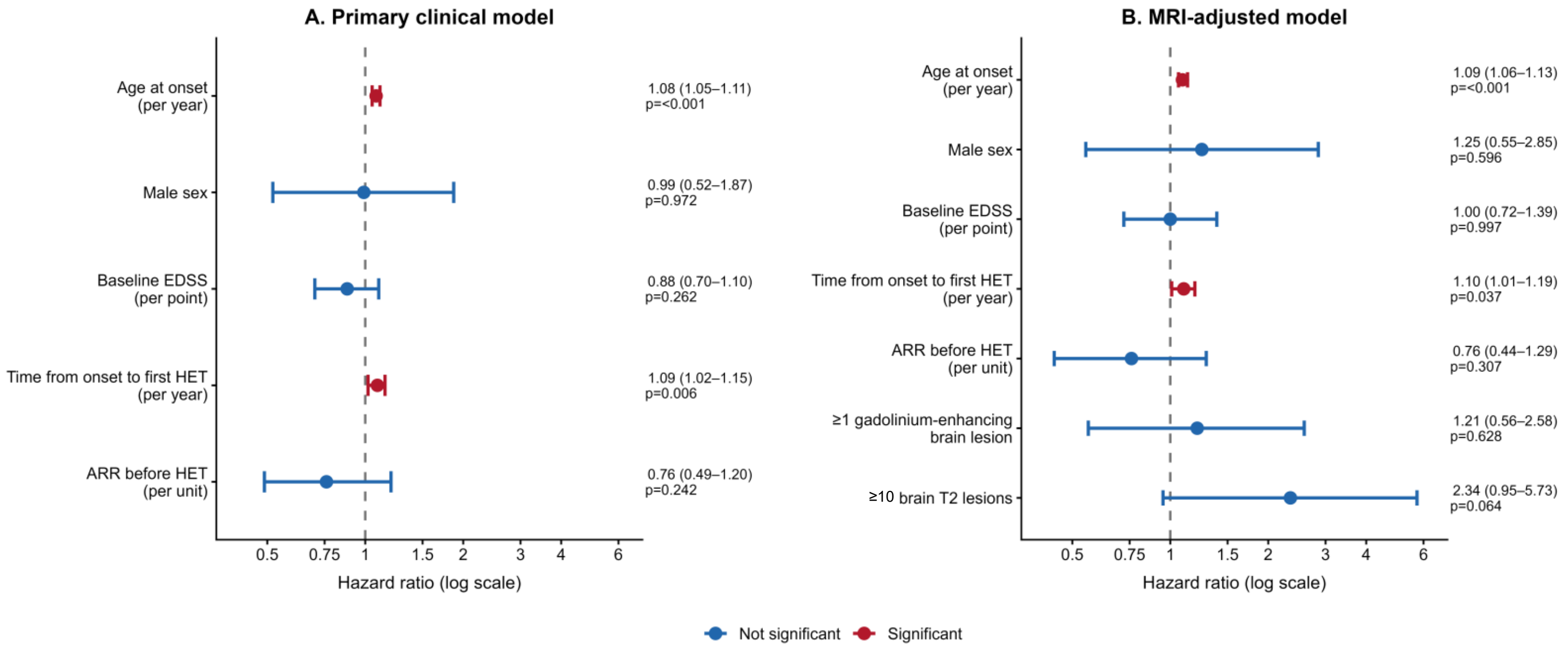

Forest plots showing hazard ratios (HRs) and 95% confidence intervals (CIs) for predictors of progression independent of relapse activity (PIRA) after initiation of a high-efficacy therapy (HET) in the on-treatment sensitivity analysis. In this analysis, follow-up was censored at discontinuation of the index HET, initiation of a subsequent disease-modifying therapy, or last available follow-up, whichever occurred first. Panel A shows the primary multivariable model including prespecified clinical variables only. Panel B shows the secondary model additionally adjusted for baseline brain MRI characteristics, including the presence of gadolinium-enhancing lesions and high T2 lesion burden (≥10 lesions). Red symbols indicate statistically significant associations (p<0.05), whereas blue symbols indicate non-significant associations. The dashed vertical line represents HR=1 (no association). Hazard ratios >1 indicate an increased risk of subsequent PIRA. Abbreviations: ARR, annualized relapse rate; CI, confidence interval; EDSS, Expanded Disability Status Scale; HET, high-efficacy therapy; HR, hazard ratio; MRI, magnetic resonance imaging; PIRA, progression independent of relapse activity.

Supplementary Figure 7. Landmark analysis with a 90-day lag period: clinical and MRI predictors of PIRA after HET exposure.

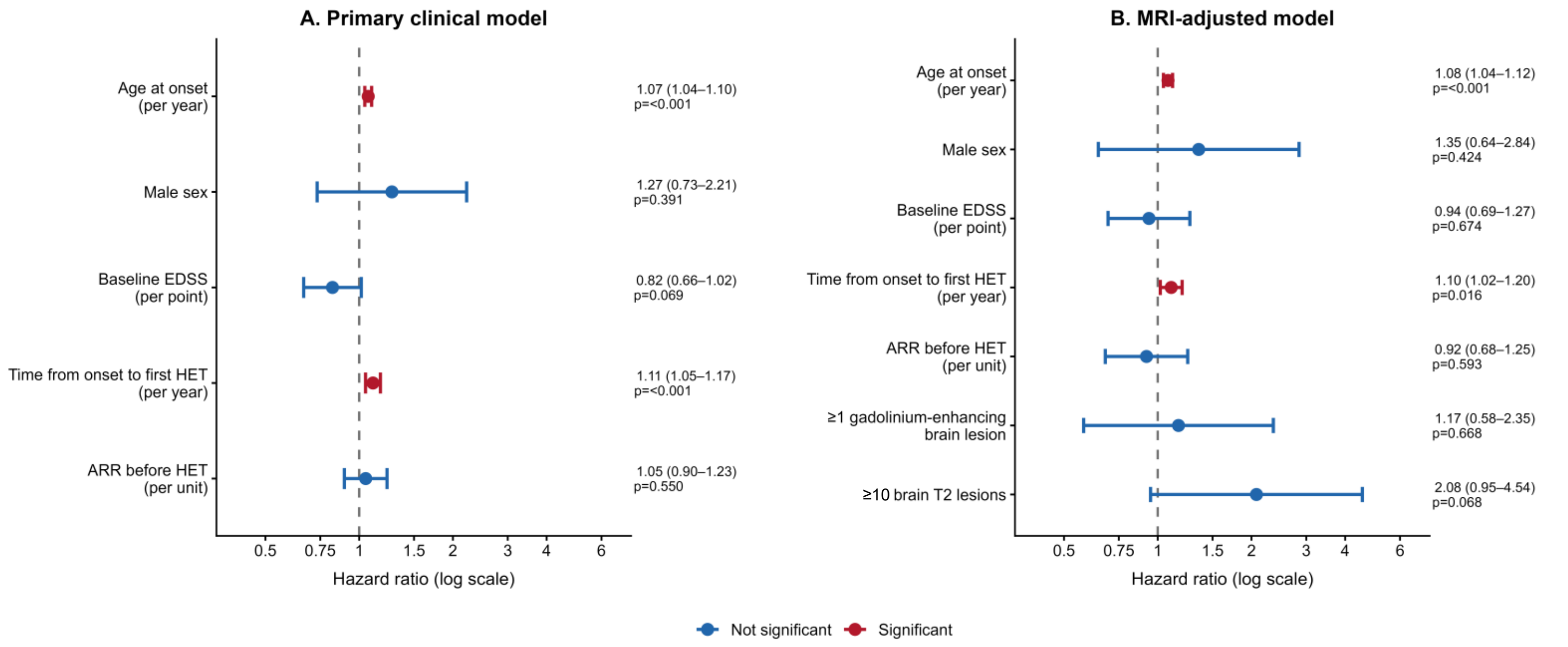

Forest plots showing hazard ratios (HRs) and 95% confidence intervals (CIs) from multivariable Cox regression models evaluating predictors of progression independent of relapse activity (PIRA) after high-efficacy therapy (HET) exposure in a 90-day landmark analysis. Patients who experienced PIRA within the first 90 days after HET initiation were excluded, and follow-up began at the landmark date (90 days after HET initiation). Panel A shows the primary multivariable model including prespecified clinical variables only. Panel B shows the secondary model additionally adjusted for baseline brain MRI characteristics, including the presence of gadolinium-enhancing lesions and high T2 lesion burden (≥10 lesions). Red symbols indicate statistically significant associations (p<0.05), whereas blue symbols indicate non-significant associations. The dashed vertical line represents HR=1 (no association). Hazard ratios >1 indicate an increased risk of subsequent PIRA. Abbreviations: ARR, annualized relapse rate; CI, confidence interval; EDSS, Expanded Disability Status Scale; HET, high-efficacy therapy; HR, hazard ratio; MRI, magnetic resonance imaging; PIRA, progression independent of relapse activity.

Supplementary Figure 8. Sensitivity analysis using EDSS re-baselined at first high-efficacy therapy initiation.

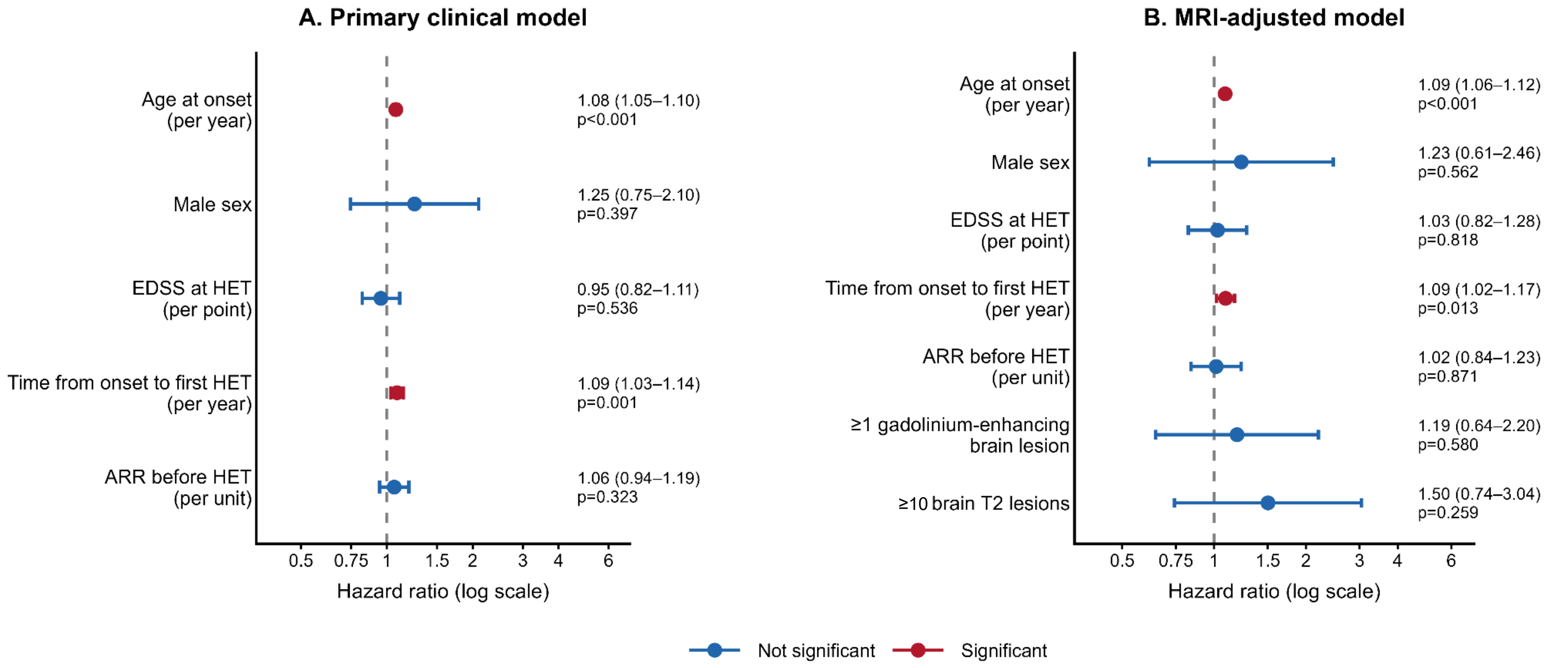

Forest plots showing hazard ratios (HRs) and 95% confidence intervals (CIs) from multivariable Cox regression models evaluating predictors of progression independent of relapse activity (PIRA) after high-efficacy therapy (HET) exposure. EDSS was re-baselined using the most recent eligible assessment obtained within 180 days before HET initiation, excluding assessments performed within 30 days after a clinical relapse, and post-HET PIRA events were re-derived using this treatment-specific baseline. Panel A shows the primary multivariable model including prespecified clinical variables only. Panel B shows the secondary model additionally adjusted for baseline brain MRI characteristics, including the presence of gadolinium-enhancing lesions and high T2 lesion burden (≥10 lesions). Red symbols indicate statistically significant associations ($p<0.05$), whereas blue symbols indicate non-significant associations. The dashed vertical line represents HR=1 (no association). Hazard ratios >1 indicate an increased risk of subsequent PIRA. Abbreviations: ARR, annualized relapse rate; CI, confidence interval; EDSS, Expanded Disability Status Scale; HET, high-efficacy therapy; HR, hazard ratio; MRI, magnetic resonance imaging; PIRA, progression independent of relapse activity.

Supplementary Figure 9. Sensitivity analysis adjusted for calendar year of first high-efficacy therapy initiation.

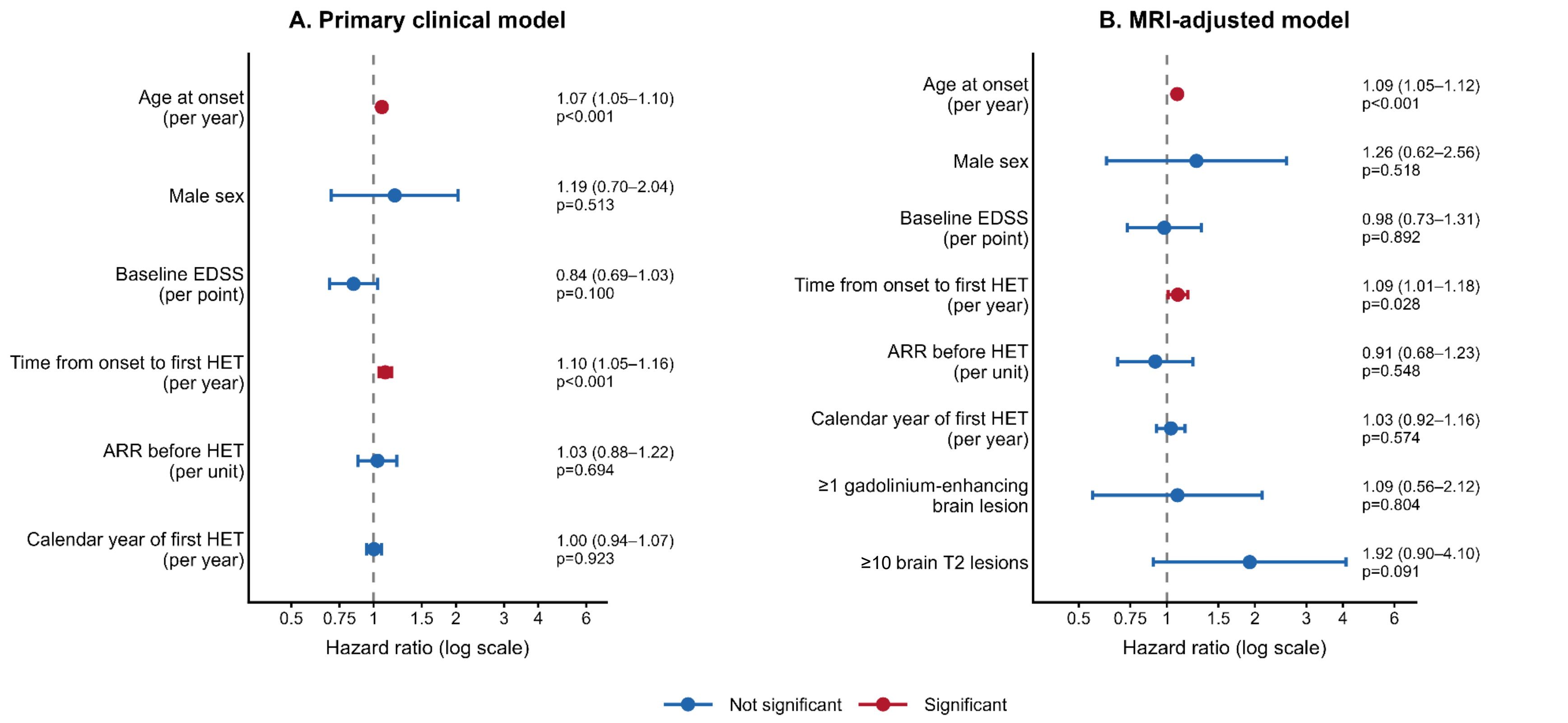

Forest plots showing hazard ratios (HRs) and 95% confidence intervals (CIs) from multivariable Cox regression models evaluating predictors of progression independent of relapse activity (PIRA) after high-efficacy therapy (HET) exposure, with additional adjustment for calendar year of first HET initiation. Calendar year was modelled as a continuous variable, with the HR representing the association per one-year increment. Panel A shows the primary multivariable model including prespecified clinical variables and calendar year of first HET initiation. Panel B shows the secondary model additionally adjusted for baseline brain MRI characteristics, including the presence of gadolinium-enhancing lesions and high T2 lesion burden (≥10 lesions). Red symbols indicate statistically significant associations (p<0.05), whereas blue symbols indicate non-significant associations. The dashed vertical line represents HR=1 (no association). Hazard ratios >1 indicate an increased risk of subsequent PIRA. Abbreviations: ARR, annualized relapse rate; CI, confidence interval; EDSS, Expanded Disability Status Scale; HET, high-efficacy therapy; HR, hazard ratio; MRI, magnetic resonance imaging; PIRA, progression independent of relapse activity.
